# Molecular Diffusion and Optical Properties of Implantable Collagen Materials

**DOI:** 10.3390/ma18051035

**Published:** 2025-02-26

**Authors:** Sofya V. Atsigeida, Daria K. Tuchina, Peter S. Timashev, Valery V. Tuchin

**Affiliations:** 1 Institute of Physics and Science Medical Center, Saratov State University, 410012 Saratov, Russia; tuchinadk@mail.ru (D.K.T.); tuchinvv@mail.ru (V.V.T.); 2 Laboratory of Biophotonics, Tomsk State University, 634050 Tomsk, Russia; 3 Institute for Regenerative Medicine, Sechenov First Moscow State Medical University, 119991 Moscow, Russia; timashev_p_s@staff.sechenov.ru; 4 Institute of Precision Mechanics and Control, Federal Research Center “Saratov Scientific Center of the Russian Academy of Sciences”, 410028 Saratov, Russia

**Keywords:** collagen, implantable materials, tissue phantom, molecular diffusion, glucose, spectral measurements, OCT

## Abstract

The effects of optical clearing of implantable collagen materials were studied using optical clearing agents (OCAs) based on aqueous glucose solutions of various concentrations. By measuring the kinetics of the collimated transmission spectra, the diffusion *D* and permeability *P* coefficients of the OCAs of collagen materials were determined as *D* = (0.22 ± 0.05) × 10^−6^ to (1.41 ± 0.05) × 10^−6^ cm^2^/c and *P* = (0.55 ± 0.04) × 10^−4^ to (1.77 ± 0.07) × 10^−4^ cm/c. Studies with optical coherence tomography (OCT) confirmed that each of the OCAs used had an effect on the optical properties of collagen materials, and allowed us to quantify the group refractive indices of the collagen of various samples, which turned out to be in the range from *n*_c_ = 1.476 to *n*_c_ = 1.579.

## 1. Introduction

Collagen is a fibrillar protein that is one of the main components of tendons, joints, bones, skin, nails, hair and teeth and also forms the walls of veins, arteries and capillaries; it can be extracted from various tissues. This type of fibrous protein accounts for approximately 25% of the total dry mass of mammals [1].

Type I collagen is a suitable material for implantation, since only a small number of people have humoral immunity against it, and a simple serological test can check whether a patient is susceptible to an allergic reaction in response to this collagen-based biomaterial [2]. It is important to note that immunogenicity is also applicable to collagen molecules that make up the extracellular matrix (ECM) and that most adverse immune reactions characteristic of ECM do not necessarily occur due to the collagen molecule itself.

There are two fundamental methods by which collagen-based biomaterials can be created [3]. The first is a decellularized collagen matrix capable of preserving the original structure of the ECM and the shape of the tissue, while the other is based on the extraction, purification and polymerization of collagen and its various modifications to form a functional framework. Both methods can be used in conjunction with various intermolecular crosslinking protocols, which makes it possible to apply them to many collagen source tissues regardless of origin.

Keane et al. considered three methods for tissue decellularization: physical, chemical and enzymatic [3]. Physical methods include rapid freezing, which induces cell lysis due to the formation of ice crystals inside cells and the high pressure they create on cell membranes. Physical methods are often used in combination with chemical methods to facilitate tissue permeability to the active molecules responsible for chemical reactions.

Chemical methods of decellularization use various reagents to remove the cellular content of ECM, for example, ethylenediaminetetraacetic acid (EDTA), ionic or nonionic detergents and solutions with extreme osmolarity. However, none of the existing methods can create ECM completely free of cellular debris, and a combination of methods is often required to obtain a material free of any cell residues. The enzymatic method uses, for example, trypsin, which specifically cleaves proteins and nucleases, removing DNA and RNA.

Another method for obtaining a collagen-based biomaterial is the extraction, purification and polymerization of collagen and its various modifications by treating a collagen solution with other biomolecules such as glycosaminoglycans (GAG) [4], elastin [5] or chitosan [6]. Modern extraction methods are based on three basic principles of solubilization: in acidic solutions [7], in neutral salt solutions [8] and in proteolytic solutions [9].

Several methods have been developed for crosslinking collagen scaffolds. These polymerization methods are divided into three types: physical, for which crosslinking occurs under the action of ultraviolet (UV) or thermal radiation inducing polymerization of the collagen framework; chemical, when crosslinking is obtained using glutaraldehyde [6,10,11], the most studied and widely used, as well as using substances from the carbodiimide family [12] or isocyanates [13]; and enzymatic, when crosslinking is formed by the action of, for example, transglutaminase and trypsin, which increase the tensile strength and enzymatic stability of a collagen-based biomaterial [5,14].

When using collagen materials as the basis for biological tissue phantoms used in optical research, in addition to various types of crosslinking, their absorbing and scattering properties are important. Optical tissue phantoms are divided into a number of types depending on how the absorption and scattering spectra of light are provided, which are close in their parameters to real tissues. If the absorption and scattering of the material does not reproduce the optical properties of the tissue well enough, then both absorbing and scattering additives are used. Typically, these are dyes and scaffold materials to reproduce the required absorption spectrum, as well as nanoparticles or microparticles with a high refractive index to reproduce scattering spectra [15]. In nanoparticle- or microparticle-based phantoms, the equivalent of the scattering coefficient is controlled by the size, shape, concentration and material of particles. Materials with intrinsic scattering are promising for the creation of phantoms because of the simplicity of their manufacture and the possibility of constructing dynamic phantoms. Such materials include porous materials based on polydimethylsiloxane and glycerol [16], an important advantage of which, in addition to controlled scattering properties, is the ability of studying the diffusion properties of tissues in relation to various metabolic molecules, such as water, glucose, glycerol, etc.

For collagen materials, their very heterogeneous structure and controlled crosslinking can provide a certain kind of transmission and reflection spectrum of the phantom, and this is the ideal case. In this sense, collagen materials are unique natural materials of animal origin, closest to the structure of real tissues [17]. They must also be permeable to metabolic and toxic molecules, which opens up the possibility of building a new type of soft tissue phantom with optical and diffusion properties close to real tissues.

Another way to create collagen-based biomaterials is three-dimensional (3D) printing, which makes it possible to create scaffolds with a suitable microarchitecture, take into account the impact of production on cell viability, and provide patient-specific spatial geometry [18].

The goal of this work is to study the kinetics of changes in the geometric and spectral properties of collagen materials during the diffusion of water and aqueous glucose solutions into them, to determine the diffusion coefficients of these molecules in collagen materials with various manufacturing technologies.

## 2. Materials and Methods

The study used collagen materials provided by the Institute of Regenerative Medicine of the I.M. Sechenov First Moscow State Medical University: “SPILAK”, “PER uncrosslinked”, “PER crosslinked” [17,19,20].

Aqueous solutions of glucose at concentrations of 20%, 32%, 40% and 50% were used as an optical clearing agent (OCA). The solutions were prepared by mixing glucose monohydrate powder (SCR Co., Ltd., Shanghai, China) and distilled water. The corresponding proportions were calculated based on the required refractive indexes of the OCA using the Gladstone–Dale relationship applicable to two-component solutions [15]:

*n*_OCA =_ *n*_w_*C*_w_ + *n*_glu_*C*_glu_, (1)

where *n*_OCA_ is the refractive index of glucose solution, *n*_w_ is the refractive index of distilled water, *C*_w_ is the volume fraction of water, *n*_glu_ is the refractive index of glucose and *C*_glu_ is the volume fraction of glucose.

The refractive indices of the glucose solutions were measured for wavelengths of 546, 589, 644, 656, 680 and 930 nm using an Abbe DR-M2/1550 (Atago, Tokyo, Japan) multiwave refractometer; the results are presented in Table 1.

The thickness of the samples, which were placed between two glass slides, was measured at 5 points using a digital two-point micrometer (FUJISAN, Fujian, China), with a measurement accuracy of 0.001 mm. The weight was measured for dry samples, then after exposure to saline and after exposure to aqueous glucose solution, using an electronic scale SA210 (SCIENTECH, West Chester, PA, USA) with an accuracy of 1 mg.

**Figure 1:**
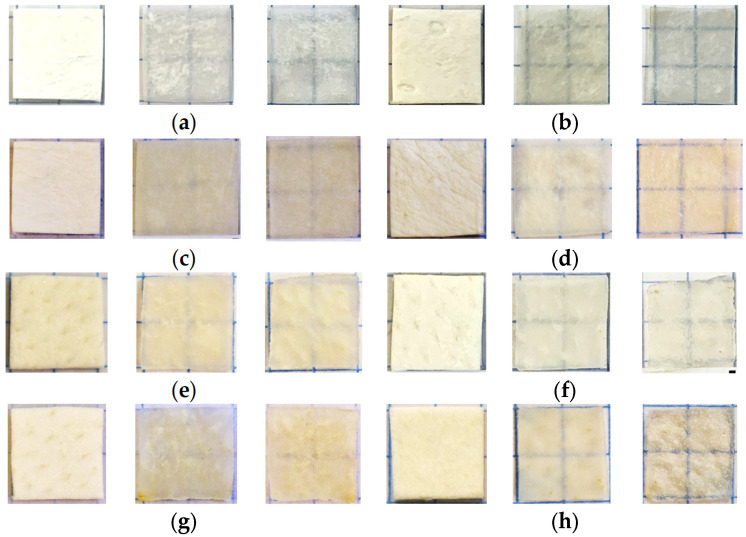
Images of the “PER uncrosslinked” (**a**–**d**) and “SPILAK” (**e**–**h**) samples in dry state (first image), after exposure to saline (second image) and after additional exposure to an aqueous glucose solution (third image) of 20% (**a**,**e**), 32% (**b**,**f**), 40% (**c**,**g**) and 50% (**d**,**h**).

Samples of about 1 × 1 cm in size were cut using a medical scalpel and surgical scissors. The prepared dry samples were incubated in a saline in a Petri dish for 30 min to simulate physiological conditions. After that, each sample was fixed on a special frame and placed in a cuvette with a prepared glucose solution. Then, sequential recording of collimated transmission spectra in the wavelength range of 500–900 nm began. The spectra were recorded at 2 min intervals for 60 min, with programmable averaging over 5 spectra. For normalization, before the start of measurements, a reference transmission spectrum of a cuvette filled with OCA with a frame but without a sample was recorded. The appearance of the samples and their transparency before and after impregnation with saline and glucose solutions can be judged by the images in Figure 1, which shows photographs of the samples placed on a substrate with blue lines drawn on it. Because of the strong scattering of dry samples, the lines were initially not visible; after impregnating the material and filling the air pores with a saline, which has a higher refractive index than air, the scattering was significantly reduced, and the samples became transparent, so the lines became distinguishable. Subsequent impregnation of the samples with glucose solutions increased the transparency and contrast of the images of these lines; the higher the glucose concentration (refractive index, see Table 1), the higher the contrast. An increase was clear in the efficiency of optical clearing for higher concentrations of glucose, as was the slightly different efficiency of clearing during impregnation with saline and subsequent saturation with glucose solution for different types of samples. For the “PER uncrosslinked” samples, the efficiency was visually presented somewhat better than for the “SPILAK” samples. For each concentration of glucose solution, 3 to 5 samples of each of the materials “PER uncrosslinked” and “SPILAK” were tested.

**Figure 2:**
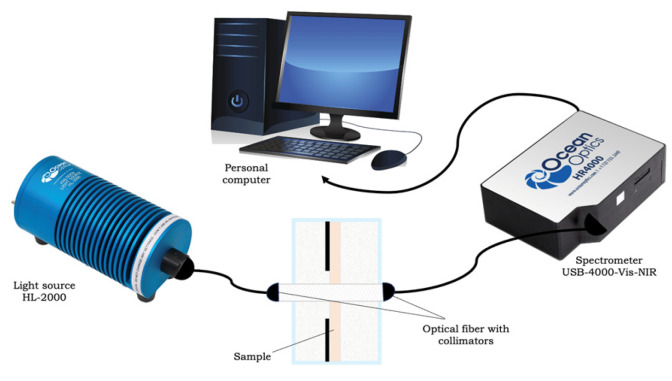
Scheme of the experimental installation.

Measurements of the optical spectra of collimated transmission of collagen samples were carried out using a USB4000-Vis-NIR multichannel spectrometer (Ocean Optics, Orlando, FL, USA). A sample of collagen material was placed in a glass cuvette with a 5 mL glucose solution mounted on a 38 × 17 (mm)^2^ frame with an 8 × 8 (mm)^2^ hole in the center. The cuvette was installed between two P400-1-UV-VIS optical fibers (Ocean Optics, USA) with a core diameter of 400 microns. To ensure the collimation of the probing beam, 74-ACR collimators (Ocean Optics, USA) were used, which produced a parallel light beam with a diameter of 5 mm on the sample at the level of 0.5 of the maximum intensity in the center of the beam. The source of the radiation was an HL-2000 halogen lamp (Ocean Optics, USA). The scheme of the experimental setup is shown in Figure 2.

The method for determining the diffusion coefficient of OCA molecules based on measurements of the kinetics of collimated optical transmission of samples *T*_c_(λ,*t*) uses the relationship between the intensity of transmitted light and the scattering coefficient, which in turn is related to the refractive index of the medium surrounding the scatterers in the sample (collagen fibers surrounded by pores). The refractive index of the medium in the pores increases because of the action of saline and OCA. Diffusion of OCA in a medium with pores filled with saline is described in the approximation of free diffusion of molecules in a homogeneous medium [15]:

*T*_c_(λ,*t*) ≈ *T*_c_(λ, *t* = 0) + (*T*_c_(λ, *t* = ∞) − *T*_c_(λ, *t* = 0)) · [1 − exp(−*t*/τ)], (2)

where λ is the wavelength of light, *t* is the current time, and τ is the diffusion time of the OCA, which is related to the diffusion coefficient defined by the following expression, valid for the case when the OCA diffuses from both sides of the sample with a thickness *l*:

*D* = *l*^2^/(π^2^τ). (3)

The permeability coefficient was determined under the assumption that the concentrations of the OCA outside the sample and inside it were equal:

*P* = *D*/*l*. (4)

To study the kinetics of changes in the weight, thickness and area of samples during immersion in saline and glucose solutions of different concentrations, the weight and thickness of each sample were measured, and photographs (digital images) were taken, first for the dry sample and then when it was immersed in the solution for 30 min.

**Figure 3:**
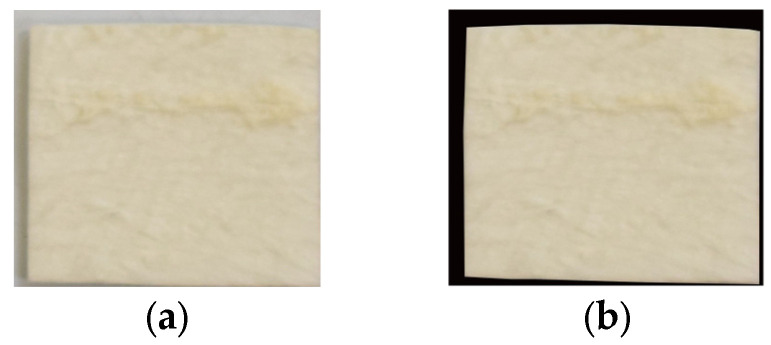
Images of “PER uncrosslinked” samples before (**a**) and after editing the image (**b**).

Figure 3 shows the principle of contactless measurement of the area of samples by photographing them and processing the images [21,22]. To correctly calculate the area of the samples, they were placed on a test object and photographed using a digital SLR camera (Nikon D5300, Bangkok, Thailand) with an image resolution of 4000 × 6000 pixels. Photographs of all samples were taken under identical conditions; the camera was mounted on a tripod with an additional light source providing uniform illumination, and the location of the tripod with the camera did not change. The images were processed in a multifunctional graphics editor (Adobe Photoshop C6), where all the samples were cut out exactly along the contour and moved to a completely black layer to get rid of all the shadows and noise that were present in any photos.

The images were then analyzed using the MathCad software (PTC, Boston, MA, USA) with the READ_READ function to decompose a full-color image (Figure 3a) into three components: red, green and blue. The median filter was used to reduce noise, eliminate glare, etc. All pixels that were not occupied by the sample were assigned the value 255 (Figure 3b). The coefficient of transition from linear dimensions in pixels to millimeters was calculated, and the size of the entire image was determined. The number of pixels occupied by the sample (with values other than 255) was calculated and converted to square millimeters using the following equation:

*S* = (*F*(*H*_S_)/(cols(*H*_S_)rows(*H*_S_)))(rows(*H*)z^2^)/cols(*H*)), (5)

where *F* is the function that calculates the number of pixels occupied by the sample; cols and rows are the number of columns and rows, respectively; *H* is the original image of the sample; *H*_s_ is the image of the sample without the background and z is the width of the sample.

**Figure 4:**
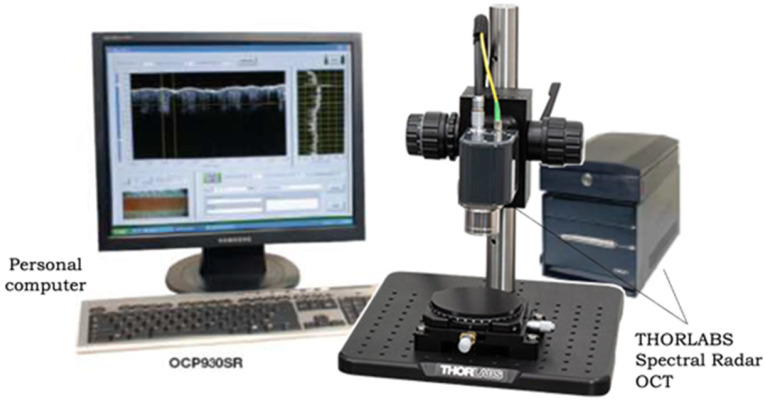
The THORLABS Spectral Radar OCT System (930 nm).

OCA diffusion in collagen materials was also monitored using a spectral optical coherence tomography (OCT) system (Spectral Radar System OCP930SR 022, Thorlabs Inc., Newton, NJ, USA) with a central wavelength of 930 ± 5 nm, a full width at half maximum (FWHM) of 100 ± 5 nm and an output power of 2 mW. The length of the longitudinal scan was 1.6 mm, with a spatial resolution of 6.2 microns in air (~4.5 microns in saline/glucose solution), and the length of the transverse scanning area along the sample surface was 2 mm, with a spatial resolution of 9.6 microns. Each B scan along the sample surface included 512 A scans. The rendering speed was 8 frames per second. The longitudinal scanning speed was ~5 kHz (Figure 4).

The OCT method was used to determine the efficiency of the optical clearing of collagen materials based on the type of dependence of the OCT signal on the probing depth for averaged A-scans. A more or less flat area was selected on the OCT image of each sample, within which 10 adjacent A-scans were selected, which were further averaged to suppress the noise component of the signal.

To determine the group refractive index of the studied “PER uncrosslinked” and “SPILAK” samples, it was necessary to determine the optical thickness of the *z*_opt_ samples from the averaged A-scans of OCT images by the distance between the reflection peaks from the front and back surfaces of the sample. The group refractive index at the central OCT wavelength λ_0_ was calculated using the expression [15]:

*n*_g_(λ_0_) = *z*_opt_/*z*_geom_, (6)

where *z*_geom_ is the geometric thickness of the sample, measured with a micrometer in microns. In order to proceed to the phase index of refraction *n*_p_ (λ_0_), it is necessary to know the dispersion of collagen material within the wavelength band of the OCT (100 nm) $\frac{{dn}_{p}}{d\lambda }{|}_{\lambda =\lambda_{0}}$ and use the following expression [23]:

(7) $$n_{p}\left(\lambda_{0}\right)=n_{g}\left(\lambda_{0}\right)+\lambda_{0}\frac{{dn}_{p}}{d\lambda }{|}_{\lambda =\lambda_{0}}$$

## 3. Results

### 3.1. Determination of OCA Diffusion Coefficients in Collagen Materials

**Figure 5:**
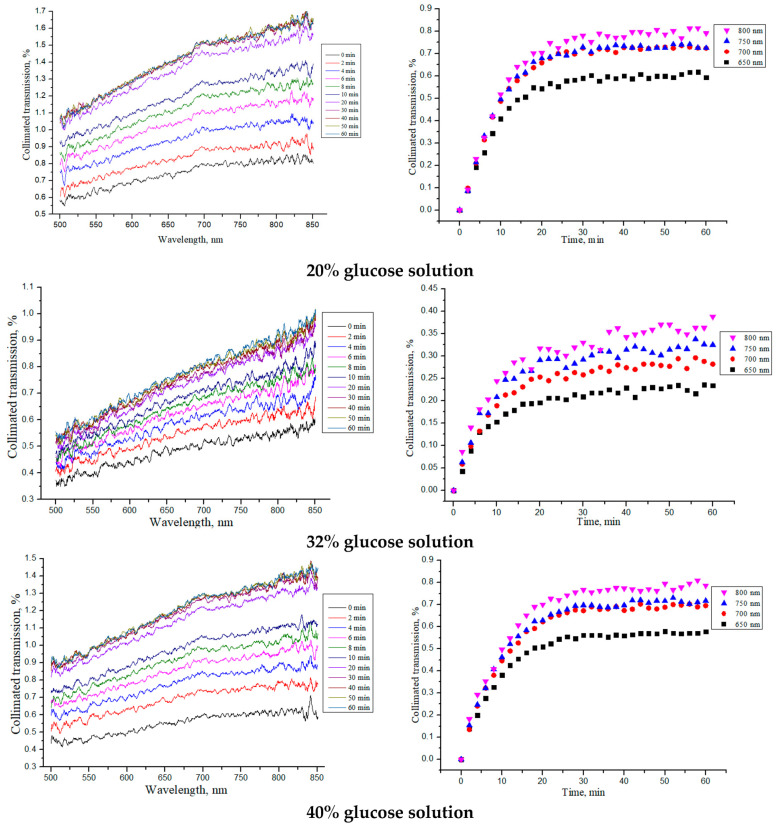

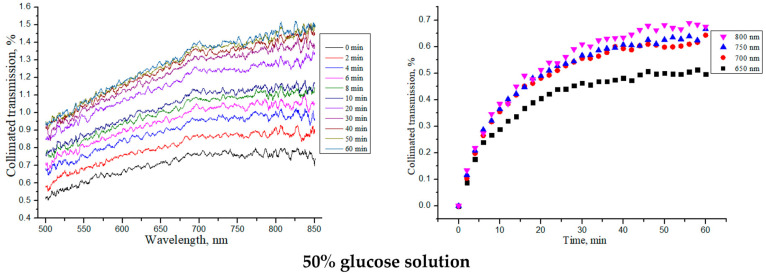
The collimated transmission spectra and the corresponding normalized kinetic dependences at wavelengths of 650, 700, 750 and 800 nm for the “PER uncrosslinked” samples under the action of aqueous glucose solutions of different concentrations.

The collimated transmission spectra and the corresponding normalized kinetic dependences at certain wavelengths (see Equation (5): (T_c_(λ, t = ∞) − T_c_(λ, t = 0)) · [1 − exp(−t/τ)]) for the “PER uncrosslinked” samples under the action of aqueous glucose solutions of different concentrations are shown in Figure 5.

Table 2 shows the average thickness of the “PER uncrosslinked” samples in the dry state, after their impregnation with saline (tissue model) and after placing saline-soaked samples in glucose solutions of different concentrations. The diffusion time *τ*, determined from the kinetic curves in Figure 5 (Equation (2)), and diffusion *D* and permeability *P* coefficients of the OCAs, calculated using Equations (3) and (4), for the sample thicknesses averaged over the entire diffusion time are also shown in Table 2.

From the obtained dependences for the collimated transmission and the values found for the diffusion and permeability coefficients, it can be seen that the rate of glucose diffusion in the “PER uncrosslinked” samples slowed down for concentrations equal to 32–40%. The thickness of the samples was significantly reduced when soaked in saline and then, when placed in glucose solution, tended to return to its initial thickness in the dry state (see Section 3.2).

**Figure 6:**
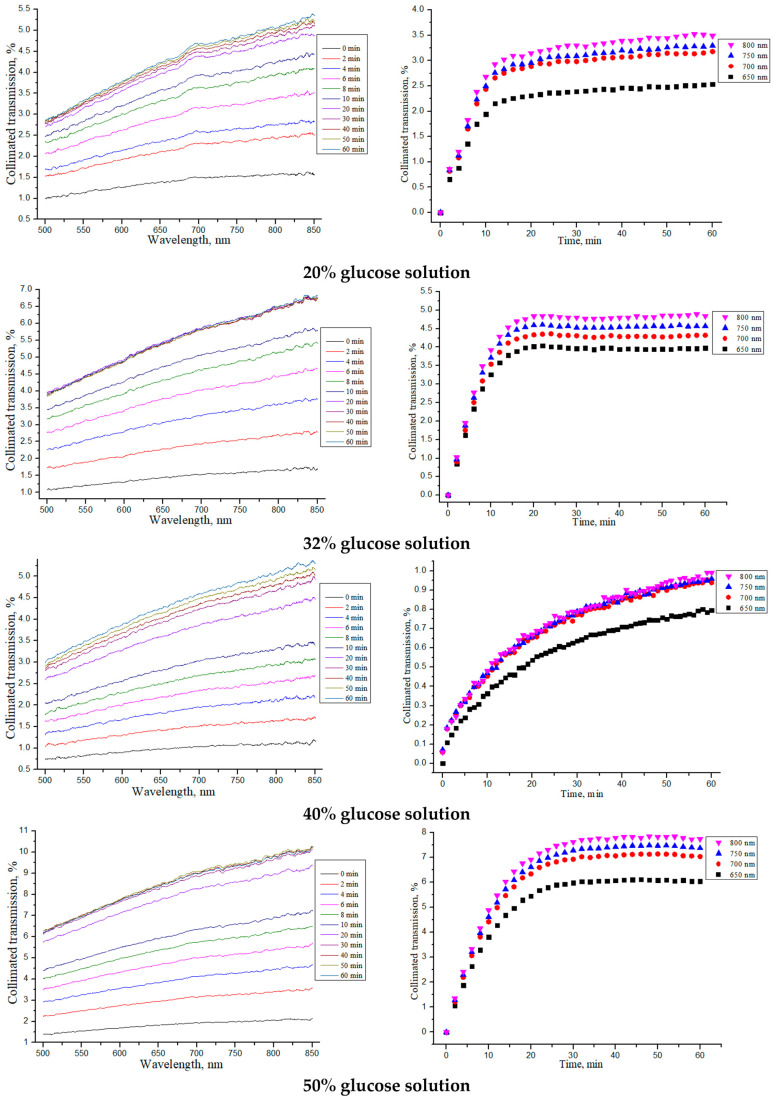
The collimated transmission spectra and the corresponding normalized kinetic dependences at wavelengths of 650, 700, 750 and 800 nm for the “SPILAK” samples under the action of aqueous glucose solutions of different concentrations.

Similar studies were carried out for the “SPILAK” samples, which are shown in Figure 6 and summarized in Table 3.

From the obtained dependences of the collimated transmission and calculated values, it can be seen that the rate of glucose diffusion in “SPILAK” samples slowed down for concentrations of 40%.

Since the samples contained large amounts of water, the diffusion coefficient of glucose in the samples should be compared with the diffusion coefficient of glucose in water. At a low glucose concentration, the glucose diffusion coefficient in water is *D*_glucose/water_ = 6.7 × 10^−6^ cm^2^/c at a temperature of 25 °C [24], which is several times higher than the estimated glucose diffusion coefficient presented in Table 2 and Table 3. The slowing down of glucose diffusion in the samples was the result of the interaction of glucose molecules with the structural components of collagen materials.

In the course of experiments, it was found that the diffusion rates of 32% and 40% aqueous glucose solutions in the “PER uncrosslinked” and “SPILAK” samples was the lowest. This was due to the achievement of an equilibrium between the water content in the sample and the solution. Under equilibrium conditions, water does not move, and only glucose diffusion occurs. Since the molecular weight of glucose is 10 times that of water, the diffusion coefficient for these concentrations was the lowest.

Saline solution has a significant effect on the optical properties of collagen materials, significantly increasing their ability to transmit light. In this context, the solution acts as an OCA for dry porous materials [16].

**Figure 7:**
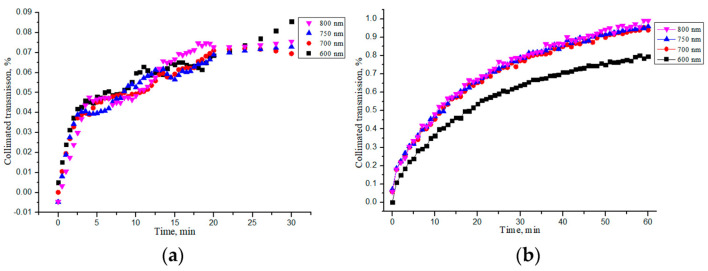
Changes in the collimated transmission of the “PER uncrosslinked” (**a**) and “SPILAK” (**b**) samples during their impregnation by saline.

The characteristic kinetic curves for the “PER uncrosslinked” and “SPILAK” samples soaked in saline are shown in Figure 7a,b, respectively. It can be noted that the increase in the collimated transmittance gradually slowed down (saturated) but did not complete during 30 min.

For ease of comparison, the average values of all quantities are given in Table 4. From these data, it is evident that the “SPILAK” samples were impregnated with saline faster than the “PER uncrosslinked” samples, which was obviously due to the different structure of these collagen materials.

### 3.2. Study of Geometric and Weight Parameters of Samples Under Saline and Water Action

**Figure 8:**
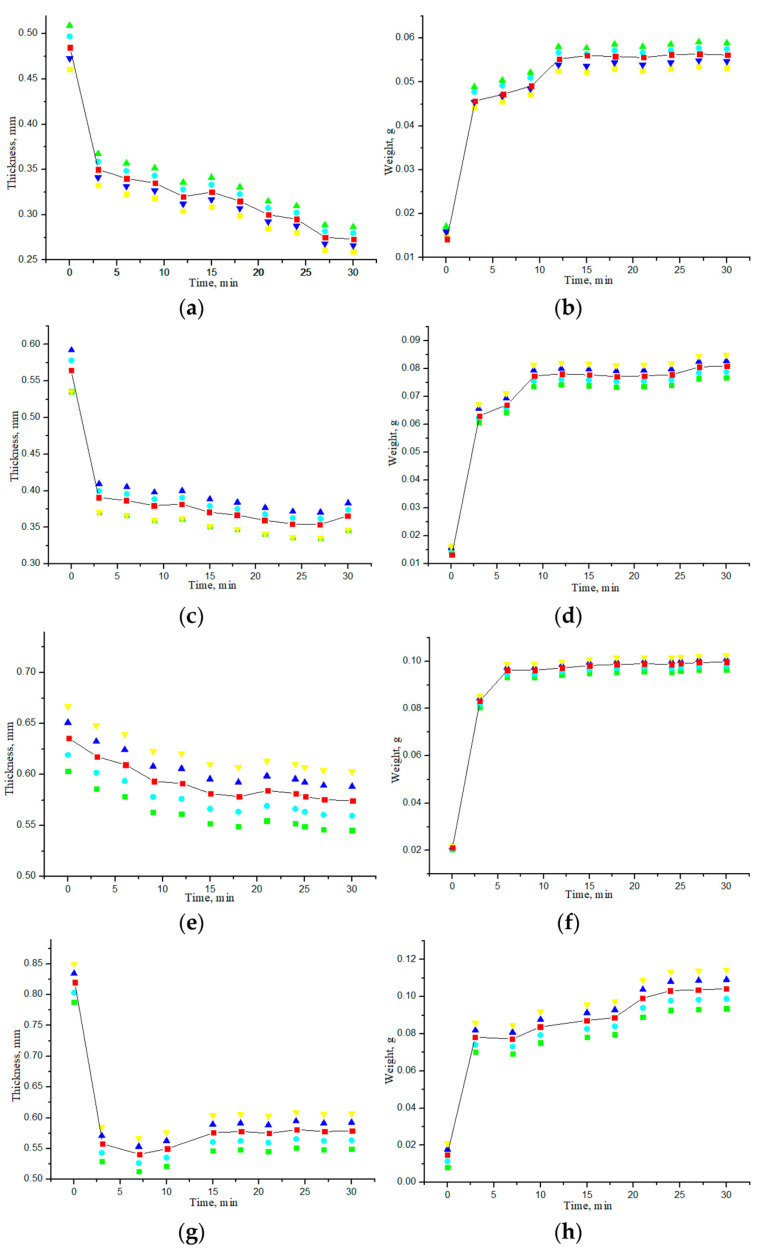

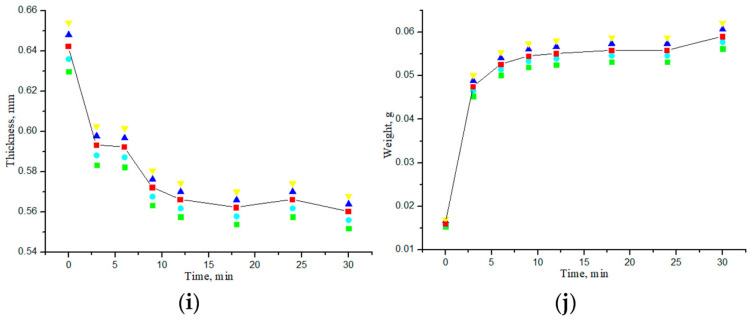
Dependences of thickness and weight of the samples on the exposure time to saline (**a**,**b**), (**e**,**f**) or deionized distilled water (**c**,**d**), (**h**,**g**), (**i**,**j**): “PER uncrosslinked” (**a**–**d**); “SPILAK” (**e**,**f**), (**h**,**g**); “PER crosslinked”(**i**,**j**).

The effect of impregnation of collagen materials with saline on the kinetics of changes in the thickness and weight of the samples was also studied. Figure 8 shows the dependences of the thickness and weight change for all samples of “PER uncrosslinked”, “SPILAK”, and “PER crosslinked” material on the time of exposure in saline or deionized distilled water. Since the samples were initially in a dry state, the increase in the mass of the samples was expected, and the detected significant decrease in the thickness of the materials impregnated with water or a saline compared with the dry ones was unexpected and is most likely explained by the fact that capillary forces act at the liquid–air interface in the porous medium of the sample during its impregnation [25,26,27,28]. When water is introduced into a porous medium, a capillary compression phenomenon may occur. It can be assumed that we are observing the capillarity effects that were previously studied in [25] in a model of one-dimensional impregnation of a deformable porous material with an incompressible fluid. The capillary rise model was formulated as a nonlinear free boundary problem, the main feature of which is that the fluid rises to a final equilibrium height, and the porous material is deformed until it reaches an equilibrium configuration that depends on the capillary pressure and the ratio of the liquid density to the material density. This phenomenon is usually associated with the process of wetting porous materials on one side, and the deformation effects are not so strong. In our work, significant deformation of soft, porous collagen sponges impregnated with incompressible liquid simultaneously from all sides was observed. When the pores were completely filled with water (saline), the OCA molecules diffused more freely in such a much more homogeneous medium.

If the material is uncrosslinked, it becomes flooded faster and loses more thickness. In control studies, the samples were impregnated with deionized distilled water purified from impurities, during which it was possible to experimentally verify that the difference in thickness between dry and water-impregnated samples was significantly smaller for crosslinked samples than for uncrosslinked ones.

The results of measuring the average thickness of each sample when exposed to a glucose solution of a certain concentration, given in Table 2 and Table 3, show that when placing the samples in an aqueous glucose solution after being in a saline, the thickness of all samples increased to varying degrees but did not reach the initial values as in dry samples. The increase in thickness, i.e., swelling, was due to a change in the structure of the porous collagen material due to the interaction of OCA with collagen. At the same time, the weight of all samples increased. The results of these measurements are presented in Table 5. This indicates that the water-filled porous structure of collagen materials is capable of being saturated with OCA. When glucose is introduced from the outside, it penetrates the pores and interacts with water molecules, forming stable hydrogen bonds, which may be related to the hygroscopicity of glucose: about 10 water molecules can join each glucose molecule [29], which can lead to a change in the properties of the sample, in particular to an increase in its mass [28,30].

A slight decrease in the weight of the “PER crosslinked” sample compared with the other samples may be associated with the formation of intramolecular and intermolecular cross-links within collagen tissue materials, which also increases resistance to collagenase cleavage [30]. Crosslinking can change the rate of degradation [31].

For comparison, the average values of the area of dry samples and samples after their impregnation with saline and subsequent OCA impregnation are shown in Table 6.

The average area of “PER uncrosslinked” samples in the dry state was 45.7 ± 2.4 mm^2^; after exposure to saline, the area increased by an average of 8.3%, and after exposure to saline/glucose solution, by 4.3%. The most significant change, up to 10%, was found for a glucose concentration of 32%, which corresponds to the most effective glucose diffusion into sample following optical clearing kinetics (Table 2). The average area of the “SPILAK” sample in the dry state was 45.5 ± 2.6 mm^2^; after exposure to saline, the area increased by an average of 7.9%, and after exposure to saline/glucose solution, it decreased by 2.7%. However, once again, the most significant increase, up to 10%, was found for a glucose concentration of 32%.

### 3.3. OCT-Monitoring of OCA Diffusion in Collagen Materials

The OCT-method with a longitudinal resolution in an aqueous medium of about 4.5 μm and a transverse resolution of 9.6 μm allows monitoring the corresponding structural inhomogeneities and, most importantly, throughout the entire depth of the sample. OCT provides information in the form of a local scattering coefficient, which is determined by the contrast of the refractive index of collagen fibers and pores filled first with air and then with water and glucose solution. The high measurement speed of OCT allows recording these structural changes almost in real time and thereby measuring the diffusion rate of molecules in a porous medium. At the same time, OCT allows measuring the thickness of the sample by reflection from the front and back boundaries of the sample.

OCT images for all samples were recorded first for dry samples, then for the same samples after exposure to saline and subsequent exposure of glucose solutions to samples already soaked in saline. This allowed us to observe changes in the structure and thickness of samples within the longitudinal and transverse resolution of OCT. A method for noninvasive assessment of molecular diffusion in tissues using OCT was proposed by Larin et al. [32]. The possibility of measuring the diffusion coefficient of glucose in tissue was demonstrated based on the analysis of changes in its scattering properties with time and penetration depth, observing the temporal evolution of the A-scan. Such an analysis of the shape of A-scans (change in slope) allowed us to quantitatively assess changes in the optical properties of samples [32,33,34,35,36,37].

**Figure 9:**
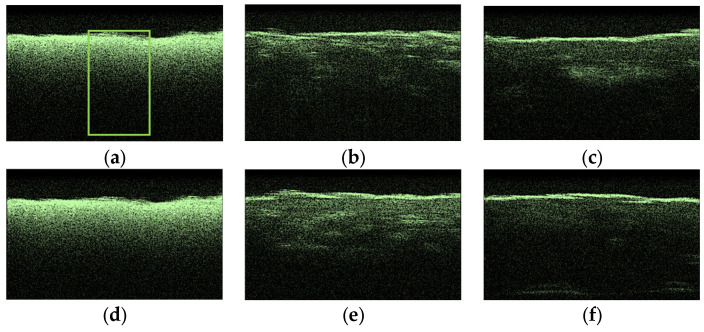
Typical OCT images of “PER uncrosslinked” samples: dry (**a**), after exposure to saline (**b**), after subsequent exposure to 20% aqueous glucose solution (**c**); dry (**d**), after exposure to saline (**e**), after subsequent exposure to 32% aqueous glucose solution (**f**).

To compare the obtained OCT images, OCA samples were selected, which showed the lowest and highest diffusion coefficients of the studied collagen materials. Figure 9 shows typical OCT images for the “PER uncrosslinked” samples for this series of experiments. The rectangle marks the area of averaging of the OCT signal (51 A-scan), which was selected in the region of interest.

**Figure 10:**
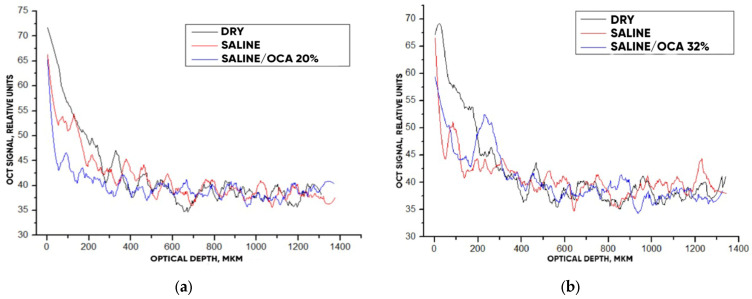
The OCT signal on the depth of “PER uncrosslinked” samples exposed to OCA: 20% (**a**) and 32% (**b**) glucose solution.

After analyzing the OCT images and averaged A-scans shown for “PER uncrosslinked” samples in Figure 9 and Figure 10, we were convinced that a 32% aqueous glucose solution was more effective than a 20% solution.

**Figure 11:**
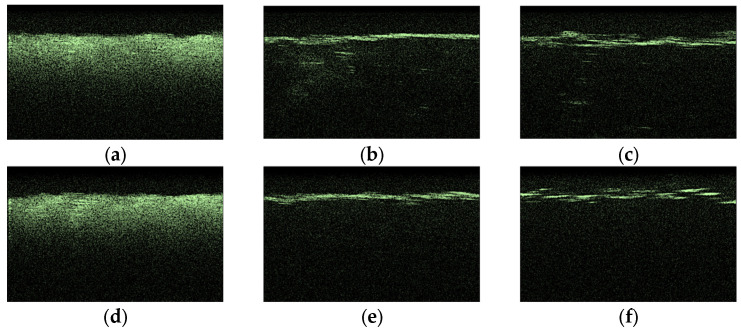
Typical OCT images of “SPILAK” samples: dry (**a**), after exposure to saline (**b**), after subsequent exposure to 40% glucose solution (**c**); dry (**d**); after exposure to saline (**e**); after subsequent exposure to 50% glucose solution (**f**).

**Figure 12:**
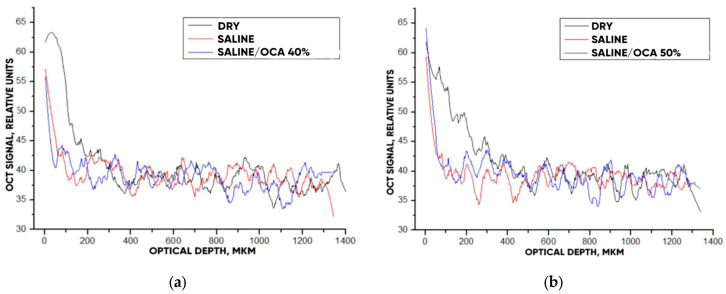
The OCT signal on the depth of the “SPILAK” samples exposed to OCA: 40% (**a**) and 50% (**b**) glucose solution.

Figure 11 hows typical OCT images for the “SPILAK” samples. From the slope of the averaged A-scans in Figure 11 and Figure 12, it can be seen that a 50% aqueous glucose solution had a greater effect on the sample than a 40% solution, which is also visible in the OCT images.

To obtain a quantitative description of the effectiveness of optical clearing of samples, a method for measuring the slope of the OCT signal dependence on depth called the OCT signal slope (OCTSS), described earlier [30,32], was used. In the first approximation, the OCTSS plotted on a logarithmic scale is proportional to the total attenuation coefficient of the tissue, μ_t_:

Ln(*I*(*z*)/*I*_0_) =^def^ OCTSS= −μ_t_*z* = −(μ_a_ + μ_s_)*z* (8)

where μ_a_ and μ_s_ are the absorption and scattering coefficients, respectively. Since μ_s_ ≫ μ_a_ in the near-infrared spectral range

Ln(*I*(z)/*I*_0_) =^def^ OCTSS= −μ_s_*z* (9)

**Figure 13:**
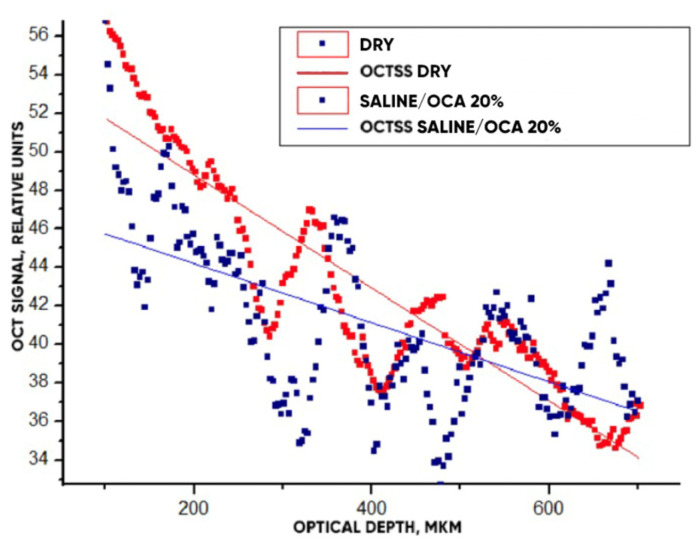
The OCT signals averaged within the window (Figure 9a) depending on the scanning depth for the “PER uncrosslinked” samples before and after the action of OCA (20% glucose) for 30 min. Straight lines present the slopes of these dependencies.

The effectiveness of optical clearing was determined as follows:

%clearning = (|*I*_2_ − *I*_1_|/*I*_1_) ∗ 100% (10)

where *I*_1_ is the optical intensity before the OCA was added and *I*_2_ is the optical intensity after the OCA penetrated to a certain depth. In our case, a range of 0.6 mm was taken. An example of calculations using the OCT signal slope method is shown in Figure 13.

The OCT signals averaged within the window (Figure 9a) depending on the scanning depth for the “PER uncrosslinked” samples before and after the action of OCA (20% glucose) for 30 min are shown in Figure 13. Approximating linear dependencies determined the slopes of the characteristics, which are directly related to the value of the scattering coefficient (see Equation (8)). A decrease in slope under the action of the OCA means that the depth-averaged scattering coefficient decreased.

The optical clearing efficiency of %clearning for “PER uncrosslinked” samples when exposed to a 20% aqueous glucose solution was 19.8%, and when exposed to a 32% solution, 28.7%. For “SPILAK” samples when exposed to a 40% solution, the optical clearing efficiency was 12.5%, and 50% solution, 21.5%.

It is important to note that in addition to increasing the intensity of the OCT signal, starting from a depth of about 500 microns, where the approximating lines intersect, the optical clearing procedure increased the contrast of images of optical inhomogeneities of the material at all depths, starting from the smallest, about 100 microns (see Figure 13).

Using OCT images, it is also possible to determine the refractive index of the studied samples from the optical thickness measured on OCT relative to real thickness measured by a micrometer. The real thicknesses were determined and are presented in Table 2 and Table 3. The optical thicknesses were calculated as the number of pixels occupied by a sample in the OCT image multiplied by the known pixel size. The average optical thickness of the saline-soaked samples was 768 ± 59 microns for the “PER uncrosslinked” and 390 ± 26 microns for the “SPILAK” materials. Using an equation similar to Equation (1),

*n*_OCT_ = *n*_c_*C*_c_ + *n*_OCA_*C*_OCA_, *C*_c_ + *C*_OCA_ = 1, (11)

where the contributions of OCA (*n*_OCA_*C*_OCA_) and hydrated collagen *n*_c_*C*_c_ to the measured group refractive index *n*_OCT_ are taken into account, it is possible to determine the refractive index of the hydrated collagen (*n*_c_) of collagen materials, which turned out to be equal to *n*_c_ = 1.476 for “PER uncrosslinked” and *n*_c_ = 1.579 for “SPILAK” materials. The values obtained are very close to the values of the refractive index of hydrated collagen in tissues, *n* = 1.474 [15].

## 4. Conclusions

It has been demonstrated that different concentrations of an aqueous glucose solution have different effects on the optical properties of collagen materials. For each type of collagen material, concentrations of an aqueous glucose solution were identified that had higher diffusion rates and greater effects on the optical clearing of the material.

In the course of experiments, the thickness, weight, and area of collagen material samples at exposure to saline and aqueous glucose solutions with different concentrations were measured.

An increase in the collimated transmission of samples of the studied materials occurred as a result of the diffusion of water and glucose molecules into the porous collagen materials. For all cases, it was characteristic that the collimated transmission of samples increased during the diffusion of saline or OCA into the sample, that is, it passed the optical clearing stage. Furthermore, after the diffusion process was completed, the kinetic curves for optical transmission were saturated, and the transmission was stably high, within the observation time. In addition, the diffusion and permeability coefficients of the collagen materials determined from kinetic dependencies were in the range *D* = (0.22 ± 0.05) × 10^−6^ to (1.41 ± 0.05) × 10^−6^ cm^2^/c and *P* = (0.55 ± 0.04) × 10^−4^ to (1.77 ± 0.07) × 10^−4^ cm/c, respectively, and close to the values typical for tissues with a high content of collagen.

With the help of OCT studies, it was confirmed that each of the OCAs used had an effect on the optical properties of collagen materials. Based on the results presented, the refractive indices of hydrated collagen were determined for the “PER uncrosslinked” samples, n_c_ =1.476, and for the “SPILAK” samples, n_c_ =1.579. The values obtained were also very close to the values of the refractive index of hydrated collagen in tissues.

All these data are important for the use of collagen materials as implantable scaffolds for regenerative medicine, as well as robust and convenient dynamic phantoms of tissues, with which it is possible to test OCAs and determine the delivery rate of drugs and metabolic agents using inexpensive optical methods.

## Figures and Tables

**Table 1:** Refractive index ***n*_OCA_** of aqueous glucose solutions at room temperature 22 °C.

λ, nm Glucose Solution, %	546	589	644	656	680	930
**20**	1.3644	1.3628	1.3611	1.3608	1.3601	1.3565
**32**	1.3778	1.3761	1.3746	1.3742	1.3733	1.3693
**40**	1.3852	1.3839	1.3821	1.3817	1.3809	1.3769
**50**	1.3997	1.3980	1.3961	1.3958	1.3950	1.3910

**Table 2:** Average thickness *l* of “PER uncrosslinked” samples in a dry state, after impregnating with saline for 30 min (tissue model) and after placing saline-soaked samples in glucose solutions (OCA) of different concentrations for 30 min, as well as the OCA diffusion time τ, diffusion *D* and permeability *P* coefficients.

Concentration of Glucose Solution	*l*, mm (Dry/Saline/OCA)	τ, min	*D*, cm^2^/c	*P*, cm/c
20%	(0.470 ± 0.146)/ (0.308 ± 0.157)/ (0.392 ± 0.07)	8.1 ± 1.2	(0.28 ± 0.12) × 10^−6^	(0.71 ± 0.07) × 10^−4^
32%	(0.343 ± 0.070)/ (0.204 ± 0.075)/ (0.304 ± 0.078)	7.6 ± 0.8	(0.18 ± 0.13) × 10^−6^	(0.61 ± 0.11) × 10^−4^
40%	(0.469 ± 0.030)/ (0.292 ± 0.050)/ (0.420 ± 0.047)	10.8 ± 1.3	(0.23 ± 0.15) × 10^−6^	(0.57 ± 0.14) × 10^−4^
50%	(0.466 ± 0.043)/ (0.287 ± 0.034)/ (0.426 ± 0.065)	11.8 ± 1.8	(0.24 ± 0.07) × 10^−6^	(0.65 ± 0.09) × 10^−4^

**Table 3:** Average thickness *l* of “SPILAK” samples in a dry state, after impregnating with saline for 30 min (tissue model) and after placing saline-soaked samples in glucose solutions (OCA) of different concentrations for 30 min, as well as the OCA diffusion time τ, diffusion *D* and permeability *P* coefficients.

Concentration of Glucose Solution	*l*, mm (Dry/Saline/OCA)	τ, min	*D*, cm^2^/c	*P*, cm/c
20%	(0.791 ± 0.153)/ (0.605 ± 0.086)/ (0.744 ± 0.087)	7.3 ± 1.4	(1.43 ± 0.41) × 10^−6^	(2.06 ± 0.22) × 10^−4^
32%	(0.831 ± 0.061)/ (0.624 ± 0.145)/ (0.766 ± 0.042)	6.9 ± 1.1	(1.52 ± 0.32) × 10^−6^	(2.32 ± 0.27) × 10^−4^
40%	(0.860 ± 0.041)/ (0.627 ± 0.077)/ (0.806 ± 0.116)	11.8 ± 2.2	(0.82 ± 0.12) × 10^−6^	(1.080 ± 0.22) × 10^−4^
50%	(0.703 ± 0.254)/ (0.472 ± 0.368)/ (0.657 ± 0.032)	8.8 ± 1.3	(1.58 ± 0.41) × 10^−6^	(2.54 ± 0.17) × 10^−4^

**Table 4:** Average diffusion time τ, diffusion *D* and permeability *P* coefficients of saline in the samples of “PER uncrosslinked” and “SPILAK” collagen materials.

Sample	τ, min	*D*, cm^2^/c	*P*, cm/c
“PER uncrosslinked”	11.9 ± 0.8	(0.22 ± 0.05) × 10^−6^	(0.55 ± 0.34) × 10^−4^
“SPILAK”	7.5 ± 0.9	(1.41 ± 0.05) × 10^−6^	(1.77 ± 0.27) × 10^−4^

**Table 5:** Average weights of dry samples and samples after impregnation with saline and subsequent impregnation with OCA (20–50% glucose solution).

Sample	Dry Weight, mg	Weight with Saline, mg	Weight with Saline/OCA, mg
“PER uncrosslinked”	14 ± 1	60 ± 5	75 ± 3
“SPILAK”	16 ± 1	83 ± 7	119 ± 9

**Table 6:** The average sample area in the dry state, after impregnation with saline and after subsequent impregnation with OCA.

	“PER Uncrosslinked”, S, mm^2^	“SPILAK”, S, mm^2^
Dry sample	45.7 ± 2.4	45.5 ± 2.6
Saline	45.6 ± 2.4	42.8 ± 2.4
20% glucose	45.3 ± 1.3	44.9 ± 6.2
32% glucose	51.6 ± 3.1	51.6 ± 2.7
40% glucose	47.1 ± 5.3	44.8 ± 8.8
50% glucose	44.4 ± 4.2	40.1 ± 5.2

## Data Availability

The original contributions presented in the study are included in the article, further inquiries can be directed to the corresponding author.

## Abbreviations

The following abbreviations are used in this manuscript:

DNA	Deoxyribonucleic acid
ECM	Extracellular matrix
EDTA	Ethylenediaminetetraacetic acid
GAG	Glycosaminoglycans
OCA	Optical clearing agent
OCT	Optical coherence tomography
OCTSS	OCT signal slope
RNA	Ribonucleic acid
UV	Ultraviolet
